# Will a new 2017 global leadership commit to NTDs?

**DOI:** 10.1371/journal.pntd.0005309

**Published:** 2017-03-23

**Authors:** Peter J. Hotez, Serap Aksoy

**Affiliations:** 1 Sabin Vaccine Institute and Texas Children’s Hospital Center for Vaccine Development, Departments of Pediatrics and Molecular Virology and Microbiology, National School of Tropical Medicine, Baylor College of Medicine, Houston, Texas, United States of America; 2 James A Baker III Institute for Public Policy, Rice University, Houston, Texas, United States of America; 3 Department of Biology, Baylor University, Waco, Texas, United States of America; 4 Scowcroft Institute of International Affairs, Bush School of Government and Public Service, Texas A&M University, College Station, Texas, United States of America; 5 Department of Epidemiology of Microbial Diseases, Yale School of Public Health, New Haven, Connecticut, United States of America; McGill University Health Centre, CANADA

By 2017 we will experience a nearly wholesale shift in global governance as it relates to the world’s neglected tropical diseases (NTDs). A new United Nations (UN) Secretary General, Antonio Guterres from Portugal, was just appointed and we’ll soon have in place a new World Health Organization (WHO) Director General. In addition, the United States Government has so far been the largest financier of NTD mass drug administration (MDA), as well as research and development (R&D) for NTDs. A new US President, President Donald Trump, is now in the White House, while Prime Minister Theresa May has been appointed as the new British Prime Minister. The United Kingdom is probably the second largest global supporter of NTDs.

How shall we advocate? What does our community of NTD scientists, public health experts, and health care providers want this new global leadership to know about our diseases? What should they prioritize? Clearly, consensus on this front is problematic, but based on your submissions, emails, and letters, here are some early thoughts.

## Continue MDA

After the launch of the Millennium Development Goals, a program of integrated MDA was proposed in *PLOS Medicine* in 2005 [1] and then implemented through financial support of the US Agency for International Development (USAID) and the British Department for International Development (DFID). According to both USAID and DFID metrics, hundreds of millions of people have received access to essential NTD medicines. The WHO has also released new metrics and process indicators on the number of people who have so far received MDA. The numbers indicate that in 2015 almost one billion people received some form of MDA (also known by the WHO as “preventive chemotherapy”), a figure that represents just over 60% of the people who require access to essential medicines for the three major soil-transmitted helminth infections—ascariasis, trichuriasis, and hookworm infection—and for schistosomiasis, lymphatic filariasis (LF), onchocerciasis, and trachoma [2]. As a result, LF, onchocerciasis, and trachoma are slated for elimination through MDA, while the World Health Assembly has also adopted a resolution to shift from morbidity prevention towards schistosomiasis elimination where feasible.

Since integrated MDA and preventive chemotherapy were launched in 2006, the number of people receiving treatment has increased steadily. However, there are concerns that financing for MDA might not continue on the same trajectory. The new DFID Minister, Right Honorable Priti Patel, has promised to scrutinize every pound of British aid [3], while USAID support has been mostly flat the last three years, hovering around US$100 million annually [4]. It remains unclear whether a new US presidential administration will continue to prioritize NTDs, and we have not seen new donor governments step up to expand this funding. However, there have been some private donations for MDA and activities through the END Fund and other organizations. Therefore, the NTD community will likely have to redouble international advocacy and education activities beginning in 2017. Fortunately, ten years ago, these MDA programs could only anticipate and promise sweeping health benefits, and now ample documentation of their global health impact is available [5], so the job of advocating for them is that much easier.

## Expanding the MDA portfolio and committing to elimination

It would also be ideal if we could expand the diseases targeted by MDA beyond the seven NTDs highlighted above. There are at least two drugs in the rapid impact package for integrated MDA—ivermectin and azithromycin—that also target scabies and yaws, respectively [6]. We have an opportunity to add these two diseases as elimination targets to those listed above. In addition, through case detection and treatment, both human African trypanosomiasis and leprosy are targeted for elimination, while guinea worm is slated for eradication [7]. Some of these programs are happening under the auspices of a 2012 London Declaration for NTDs [7]. There are also further opportunities to link NTD elimination efforts with programs targeting HIV/AIDS, tuberculosis, and malaria [8]. However, increased government financing will need to be committed by the group of seven (G7) countries for this to happen.

As an alternative, since it has been pointed out that most of the NTDs and other poverty-related neglected diseases are found among the poor in the group of 20 (G20) nations, NTD elimination could be prioritized for a future G20 summit [9]. In so doing, it would become possible to expand commitments by new state actors—including Brazil, Russia, India, China, and South Africa (BRICS) nations and other G20 countries, such as Japan, South Korea, and Saudi Arabia—to global NTD elimination efforts. Placing NTDs on the G20 agenda could become an important activity by the US Department of State and the British Foreign Ministry or DFID.

## Expand the R&D agenda—New tools and basic research

There also remains an urgent need to expand the R&D agenda for NTDs. Both the 2014 Ebola outbreaks in West Africa and the 2015 to 2016 Zika epidemics in the Western Hemisphere highlighted a mostly empty pipeline of new NTD products. Together with the leaders of the major nonprofit product development partnerships (PDPs) and WHO Special Programme for Research and Training in Tropical Diseases (TDR), we previously identified a number of new drugs, diagnostics, vaccines, and vector control technologies that will be required to achieve elimination for all of the 18 NTDs prioritized by the WHO [10]. Once again, it is unlikely that we can rely exclusively on the US and UK governments to pay for such an R&D agenda. A new initiative known as the Coalition for Epidemic Preparedness Innovation (CEPI) is being formed to finance new vaccines for pandemic threats, but it remains unclear whether this effort would also address chronic and debilitating NTDs. Most of the G20 nations have advanced biotechnology capabilities, but so far there has not been a major push to redirect those activities specifically towards NTD technologies [9]. Important exceptions include recent efforts by the Governments of Japan, Germany, and the Netherlands, as well as the European Union (EU) to support global NTD R&D [9]. Again, pressure by the US and UK leadership through global health and science diplomacy efforts could help to ensure that all of the G20 nations participate. On the US side, such activities could include an expanded role for the US Science Envoy program that was created in 2009 [11]. Still another option is using US and UK leadership to spur private philanthropic investment in the NTDs, along the lines of the Chan-Zuckerberg US$45 billion launch to link biomedical science with engineering [12].

## Concluding statement

At the start of 2017 we will see new leadership at the UN, WHO, and the US and UK governments. Together with the World Bank and other UN agencies, there will be a need to regroup, refocus, and redouble global efforts towards NTD elimination. A multipronged and multinational approach will be required that includes expanding MDA, advancing additional disease elimination targets, and creating an ambitious R&D agenda. So far, the US and UK governments together with the governments of Japan, Germany, the Netherlands, and the EU have been mostly alone in providing external funds for the NTD programs. This situation must change to a broader G20 remit, hopefully through an expansion in NTD support and activities initiated by the new global leaders in 2017.
